# Supplementary material for: Closing the Gap in Surveillance and Audit of Invasive Mold Diseases for Antifungal Stewardship Using Machine Learning
Source: J Clin Med. 2019 Sep 5;8(9):1390. doi: 10.3390/jcm8091390 (PMC6780614; doi:10.3390/jcm8091390)
Supplement: Supplementary file 1 [file jcm-08-01390-s001.pdf]

Supplementary material

Table S1. A sample of 10 anonymised chest CT reports with NLP probabilistic and binary outputs, where a binary output of 1 denotes "possible fungal" for verification using medical record review.

Patient no.	Procedure	Report text	NLP Fungal probability	Binary prediction (1=possible fungal for further review, 0=else)
1	CT Chest	CT Chest performed on XXXX: Clinical notes: External CT abdo found ectatic fluid filled tubular structure in RLL and pleural based opacity within lingula . ?Aetiology. PHx CMML, GVHD, immunosuppressed. Technique: Post-contrast CT chest. Comparison: Radiograph from XXXX and CT chest from XXXX. Findings: Bronchocentric consolidation, centrilobular nodules and probable mucus plugging is present in the right lower lobe (lateral basal segment) and left upper lobe (superior lingula segment). The left upper lobe consolidative changes become confluent in the periphery with some additional ground-glass change. Mild bronchiectasis in the posterobasal segment of the lower lobes. No pleural effusion. Prominent mediastinal and bilateral hilar lymph nodes are likely reactive. Small hiatus hernia. No pericardial effusion. Flowing ossification along the right anterolateral aspects of the mid-lower thoracic vertebral bodies, consistent with DISH. Hazy increased attenuation of the small bowel mesentery (non-specific). Conclusion: Multifocal areas of consolidation and likely also mucous plugging in both lungs. The imaging findings are non-specific although infection is favoured, particularly in the setting of the patient's immunosuppression. Given the lack of significant respiratory compromise, fungal organisms require consideration (bacterial organisms thought less likely). Follow-up to radiographic resolution recommended.	0.81227958	1
2	CT Ch-Abdo-Pel-HR Chest	CT Abdomen Pelvis and High Res Chest performed on XXXX: Indication: XXyo female persistent febrile neutropaenia. ?Occult source ?Invasive fungal or opportunistic infection. Technique: Non contrast helical acquisitions through the chest, abdomen pelvis. IV Omnipaque 350 administered. Findings: Comparison with the CT chest dated XXXX and CT abdomen on XXXX. Chest: Interval development, of diffuse ground-glass opacity throughout the upper lobes and apical segments of the lower lobes. There is some associated peripheral interlobular septal thickening. A few scattered semi-solid nodules are seen bilaterally, but this is not the predominant process. No large area of consolidation or cavitatory lesion. Mild dependent atelectasis. Small bilateral pleural effusions stable. Multiple mediastinal lymph nodes, stable in size and likely reactive. Trace pericardial fluid. Abdomen and pelvis: Perinephric stranding has progressed, consistent with medical renal disease. Remaining intra-abdominal appearances are stable, and unremarkable within	0.62609029	1

		the limits of non-contrast scan. Conclusion: Interval development of diffuse ground-glass infiltrates, predominantly affecting the upper lobes. Infective aetiology is thought most likely in this clinical setting, however pulmonary oedema remains in the differential. Opportunistic infection should be excluded.		
3	CT Chest High Res	CT High Resolution Chest performed on XXXX. Findings: Longstanding elevation of the left hemidiaphragm. Bronchial wall thickening, small airway dilatation and extensive mucous plugging within the right lower and middle lobe segmental bronchi. Developing bronchopneumonia right lower lobe. No pulmonary nodules or ground glass opacities. Bilateral dependent pleural effusions. No mediastinal or hilar lymphadenopathy. No pericardial effusion. Conclusions: Small airways bronchiolitis, mucous plugging and evolving right lower lobe bronchopneumonic consolidation. The findings favour an infectious aetiology such as a gram-negative pneumonia. Pulmonary GVHD cannot be assessed in the setting of an acute pneumonia.	0.56416035	1
4	CT Chest High Res	CT High Resolution Chest performed on XXXX: Indication: Febrile neutropaenia, persisent fevers, persistent ?bronchopneumonia on imaging ?fungal infection. Technique: Routine unenhanced HRCT protocol. Findings: Comparison with XXXX. Background centrilobular emphysema. Interval development of multifocal consolidative lesions within the left lung. These are seen in the left apex, (significantly progressed since the prior study), the posteromedial and posterolateral lower lobe and the lingula. The latter three lesions are new. All lesions demonstrate dense consolidation, some with airbronchograms, but no definite central cavitation. The left lower lobe posterolateral lesion has a ground glass halo, and there is suspicion for developing ground glass around the other lesions. A tiny focus of developing consolidation in the apical left lower lobe is also new. Bibasal changes of bronchopneumonia are stable. New small bilateral pleural effusions. Calcified mediastinal lymph nodes again noted. Conclusion: Progressive multifocal consolidation involving the left lung in keeping with progressive infection. Atypical organisms must be excluded, in particular fungal infection. The presence of a ground glass halo raises the possibility of invasive aspergillous.	0.89609259	1
5	CT Pulmonary Angio	CT Angiogram performed on XXXX: CT contrast was administered CT Pulmonary Angiogram performed on XXXX: Indication: XX F with progressive multiple myeloma. Pleuritic chest pain for 5/7 (L lower ribs) and decreased exercise tolerance. Exclude PE. Findings: Comparison with the previous PET CT dated XXXX and the recent plain radiographs. Adequate contrast opacification of pulmonary arterial tree. No filling defect to suggest pulmonary embolus to the subsegmental level. Significant increase in volume of bilateral large pleural effusions with near complete compressive atelectasis of the left lower lobe and partial atelectasis of the right lower lobe. No focal pulmonary nodule. Multiple bilateral lucent/expansile rib lesions, with adjacent nodular soft tissue, progressed since the PET CT. Healing/healed pathologic fracture of the right third rib stable. Conclusion: Increase in volume of large bilateral pleural effusions with compressive atelectasis of the lower lobes. No PE. Multiple bilateral subtle rib lesions with adjacent soft tissue nodularity in keeping with myelomatous deposits, increased since the recent PET CT in XXXX	0.53603923	1

6	CT Chest High Res	CT High Resolution Chest performed on XXXX: Indication: Septic deterioration. In context of prolonged neutropenia on broad spectrum Abx. ? New fungal lung disease. Technique: Standard HRCT protocol. Findings: Left PICC in situ, tip at the atriocaval junction. Small volume wedge-shaped consolidation involving the posterior segment of the left upper lobe, with adjacent scattered nodules, in keeping with infective change. Ill-defined ground glass opacity and sub 5-mm peribronchial nodules posterior segment of the right upper lobe, also in keeping with infective/inflammatory change. No cavitatory lesions. Mild bilateral dependent atelectasis. No bronchiectasis . Trace right pleural fluid. No air trapping on expiratory scans. Conclusion: Multifocal infective changes involving the posterior segments of both upper lobes, with confluent consolidation within the left upper lobe. Radiographic follow up to imaging resolution recommended.	0.78129905	1
6	CT Chest	CT Chest performed on XXXX: CT contrast was administered Technique: Post intravenous contrast enhanced study. Findings: Comparison has been made to the previous studies including the most recent CT chest/pulmonary angiogram performed XXXX. PICC line in situ via a left-sided approach with the tip at the atriocaval junction. There are no enlarged axillary lymph nodes. Subcentimetre mediastinal and hilar nodes. The bilateral lung parenchymal consolidation within the left upper lobe and right middle lobes is less confluent. Further patchy areas of nodular opacity within the remainder of the lobes. There is no cavitation. There is the interval development of a small volume right pleural fluid. No left pleural fluid. Given the limitations of the contrast timing, the visualised portions of the upper abdominal solid viscera have a normal appearance. Comments: Radiologically improved bilateral consolidation/bronchopneumonia. Interval development of a small volume right pleural fluid.	0.49617958	0
7	CT Chest High Res	CT High Resolution Chest performed on XXXX: Clinical history: XXF with ongoing fevers/hypotension on b/g of AML (neutropenic)- ? collection / colitis Technique: Findings: Comparison with CT Chest XXXX Resolving diffuse peribronchial nodules with reduction in both the size and number of nodules. Persistent peri-nodular ground glass opacities, such as adjacent to the right basal nodules, raises the possibility of fungal etiology. A few more confluent consolidative foci, such as in the middle lobe, have likewise decreased. No cavitary nodule. No pleural effusion. No mediastinal or hilar lymphadenopathy. Minimal anterior pericardial thickening / effusion, which is improving. The density of the blood pool is in keeping with anaemia. Visualized upper abdomen is unremarkable. No suspicious osseous erosion. Conclusion: Improving peribronchial nodules consistent with resolving infection. The differential diagnosis includes atypical infection, in particular fungal organisms.	0.71393764	1
8	CT Chest High Res	CT High Resolution Chest performed on XXXX Clinical history: XXM AML on immunotherapy. PHx fungal lung infection. New R) pneumonia. Persistent hypoxia + decreased AE R) lung. ? Extent infection / effusion / fungal disease / pneumonitis secondary immunotherapy Technique: Unenhanced high-resolution CT Chest Findings: Comparison with CT Chest XXXX. Progression of ground-glass opacities in the right lung apex, which has a geographic appearance with adjacent	0.7103979	1

		reticulation. New lobular ground-glass opacity left lung apex and diffuse bilateral new ground-glass nodules, predominately within the right lung. No cavitary nodule. Atelectasis right middle lobe and posterobasal right lower lobe. Diffuse bronchial wall thickening without bronchiectasis. No pleural effusion. No mediastinal or hilar lymphadenopathy. Visualised upper abdomen is within normal limits. No suspicious osseous erosion. Superior end plate depression with sclerosis T3, T5, T6 and T11 are longstanding. Conclusion: Over appearances consistent with progression of fungal disease with a differential diagnosis of atypical infection (e.g. viral pneumonia). No pleural effusion.		
9	CT Chest High Res	CT High Resolution Chest performed on XXXX: Clinical history: XXF Febrile neutropenia. Clinical sinusitis. Increasing nodules on XR Technique: Unenhanced CT Chest Findings: Comparison with CT Chest XXXX. No lung nodule, mass or confluent consolidation. No pleural effusion. No mediastinal or hilar lymphadenopathy. Visualised upper abdomen is within normal limits. A few calcified granulomas are noted within the right lobe of liver, unchanged from previous. No suspicious osseous erosion. Both pectoral muscles appear now within normal limits with no evidence of hematoma, mass or asymmetric thickening. Conclusion: No evidence of lung nodules or opportunistic lung infection. Interval resolution of left pectoral mass.	0.28507653	0

CT, chest tomography; NLP, natural language processing.

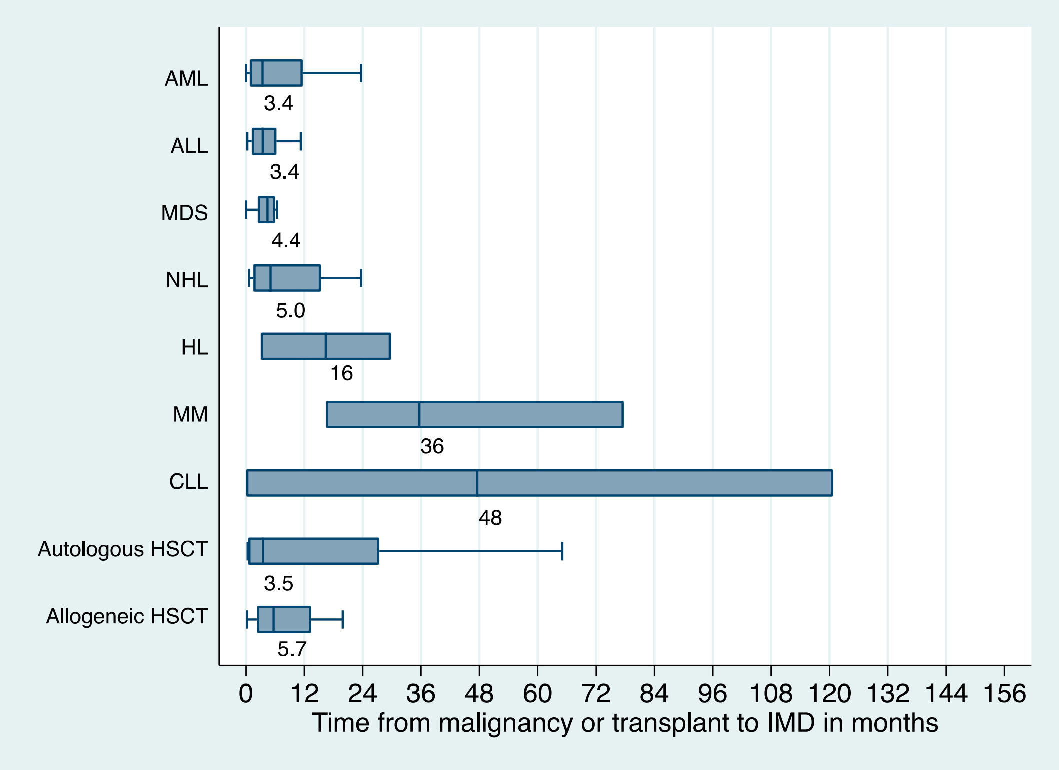

Figure S1: Time to invasive mold disease from diagnosis of haematologic condition

The boxes represent IQR 25th/75th percentiles, horizontal lines within the box represent the medians for each group, and the lines extending from the boxes represent the ranges of the data (excluding outliers). Median values are shown on the graph. Abbreviations: AML, acute myeloid leukaemia; ALL, acute lymphoblastic leukaemia; NHL, non-Hodgkin's lymphoma; MM, multiple myeloma; CLL, chronic lymphocytic leukaemia; HSCT haemopoietic stem cell transplant.

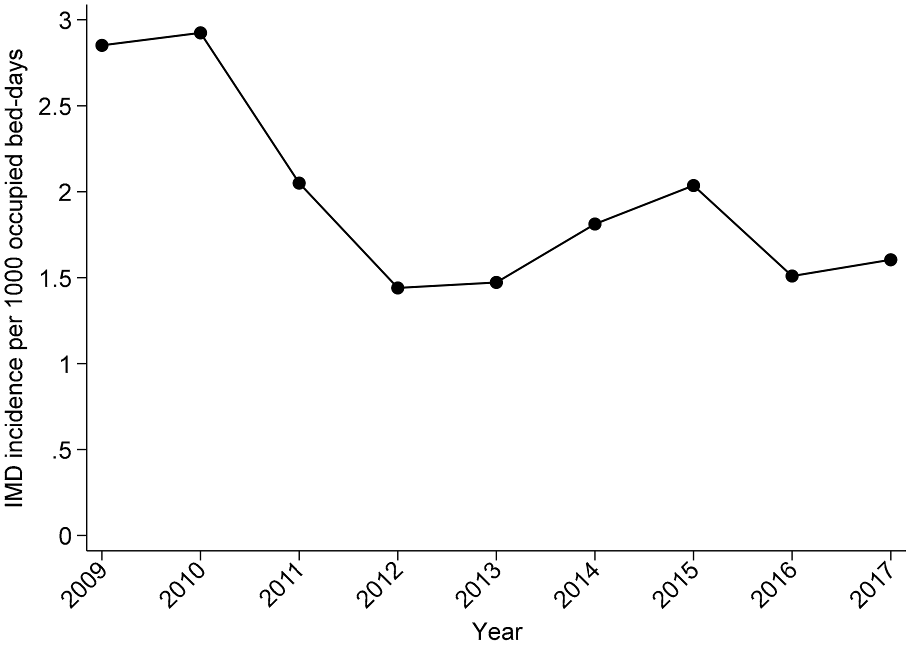

Figure S2. Incidence of invasive mold disease (IMD) 2009-2017*.

* Decreasing trend, p= 0.006

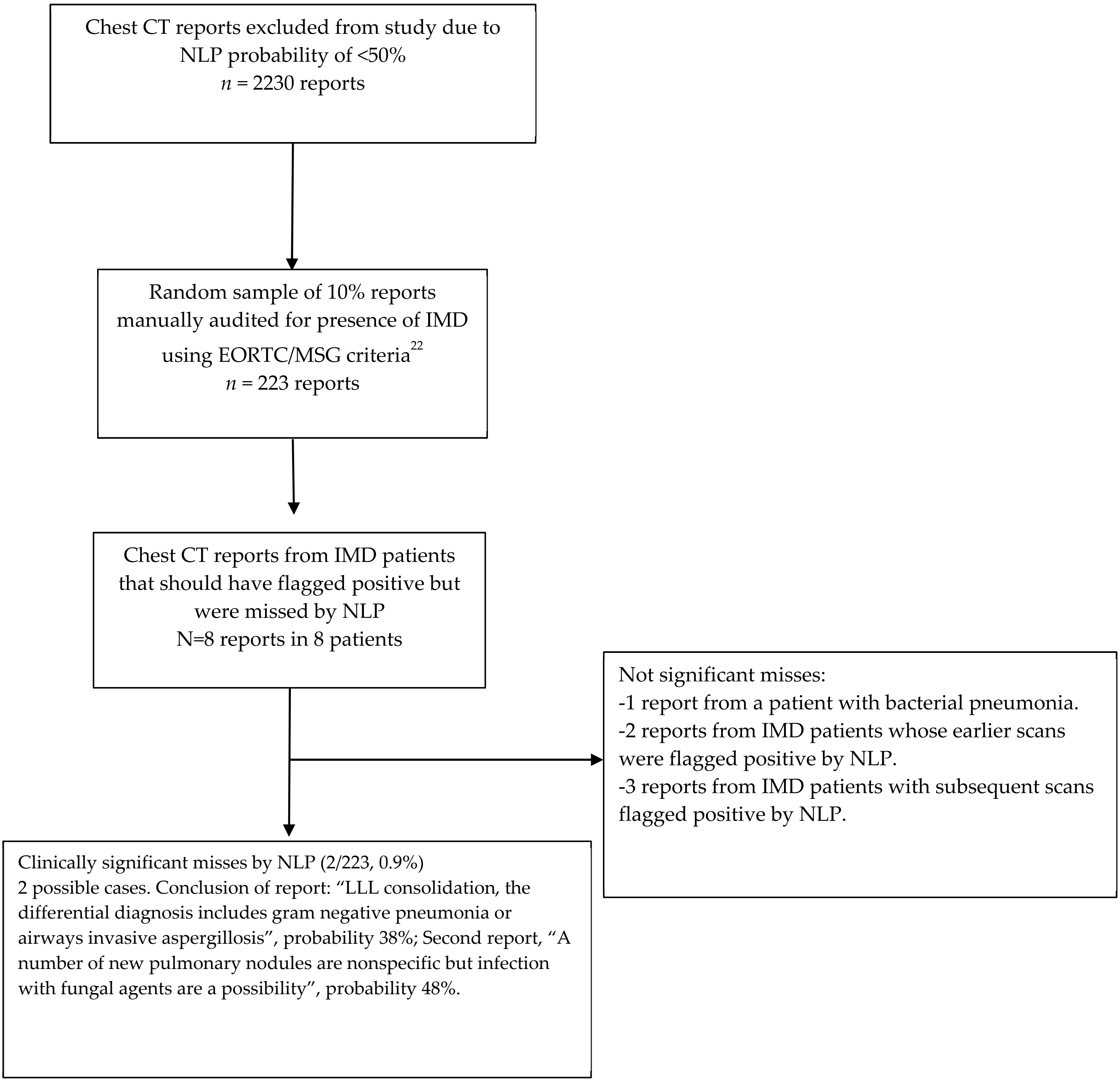

Figure S3. Random audit of 10% of chest computed tomography reports screened negative by natural language processing (NLP).

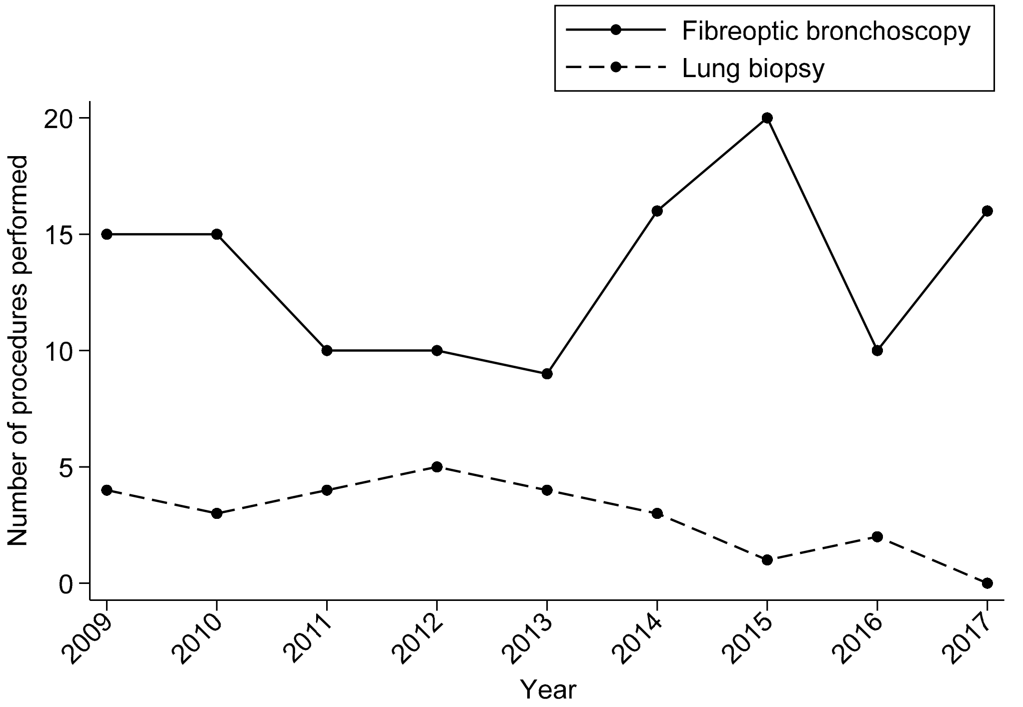

Figure S4. Number of fibreoptic bronchoscopies (total N=121) and lung biopsies (total N=26) performed per year.

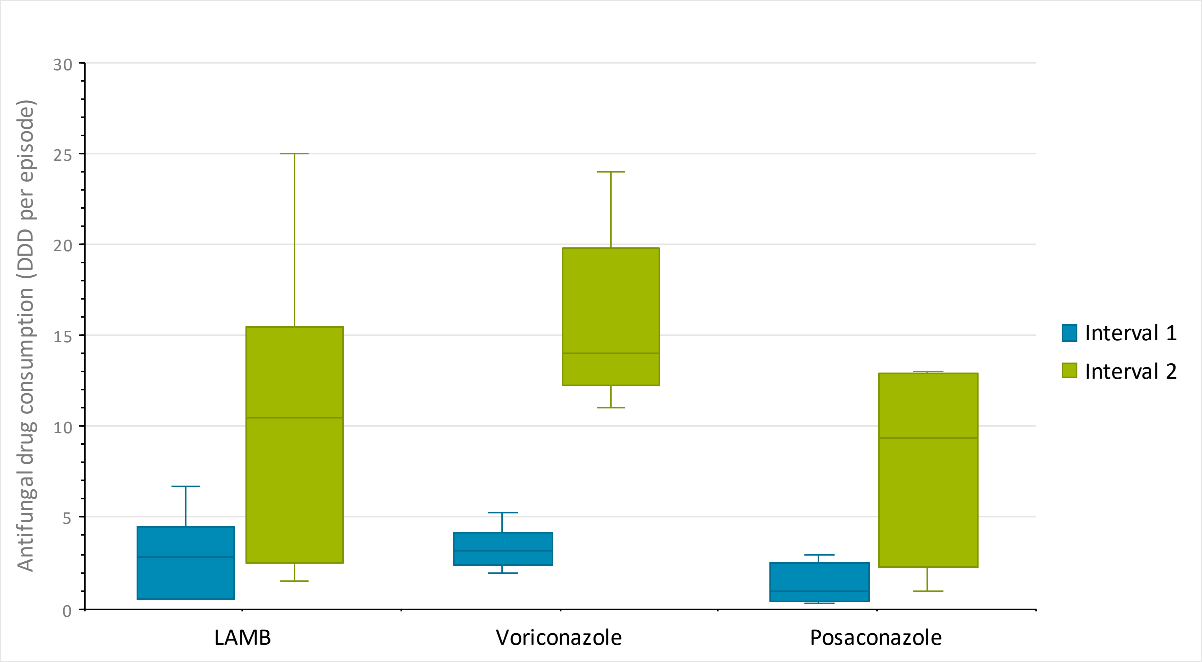

Figure S5. Antifungal drug consumption in probable IMD episodes where diagnosis made with positive bronchoalveolar lavage galactomannan only, N=19 episodes.

Interval 1 is time from CT scan to fibreoptic bronchoscopy. Interval 2 is time from fibreoptic bronchoscopy to galactomannan result reflecting turnaround time of send away testing. The boxes represent IQR 25th/75th percentiles, horizontal lines within the box represent the medians for each group, and the lines extending from the boxes represent the ranges of the data. Abbreviations: DDD; defined daily dosage

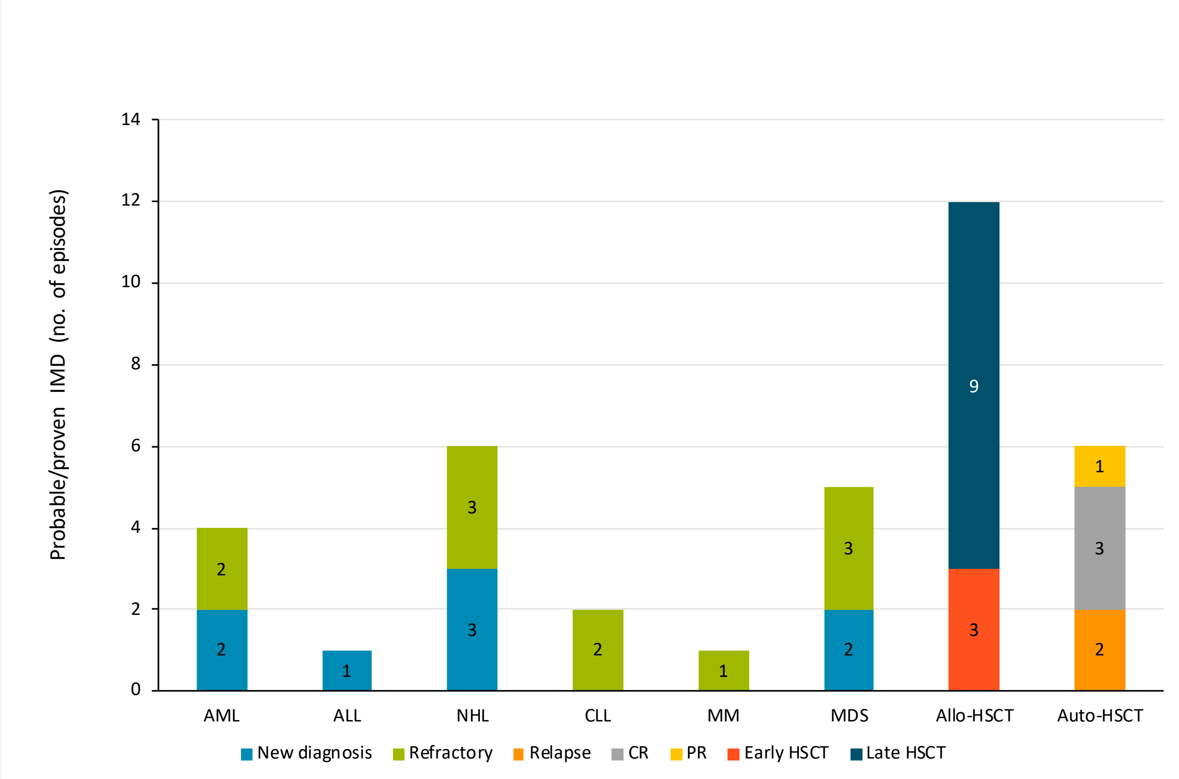

Abbreviations: AML, acute myeloid leukaemia; ALL, acute lymphoblastic leukaemia; NHL, non-Hodgkin's lymphoma; CLL, chronic lymphocytic leukaemia; MM, multiple myeloma; HSCT haemopoietic stem cell transplant (allo, allogeneic; auto, autologous); CR, complete remission; PR, partial remission.

Figure S6. Probable/proven IMD episodes (total N=35) where antifungal prophylaxis was not administered in the 14 days prior to IMD diagnosis.
